# Supplementary material for: Graphene Oxide Nanoplatforms to Enhance Cisplatin-Based Drug Delivery in Anticancer Therapy
Source: Nanomaterials (Basel). 2022 Jul 11;12(14):2372. doi: 10.3390/nano12142372 (PMC9321599; doi:10.3390/nano12142372)
Supplement: Supplementary file 1 [file nanomaterials-12-02372-s001.zip › nanomaterials-1804552-supplementary.pdf]

Supplementary Materials

Graphene Oxide Nanoplatforms to Enhance Cisplatin-Based Drug Delivery in Anticancer Therapy

Elena Giusto [1,†], Ludmila Žárská [2,†], Darren Fergal Beirne [3], Arianna Rossi [1,4], Giada Bassi [1,5], Andrea Ruffini [1], Monica Montesi [1], Diego Montagner [3,*], Vaclav Ranc [2,6,*] and Silvia Panseri [1,*]

1 Institute of Science and Technology for Ceramics–National Research Council (CNR), 48018 Faenza (RA) Italy; elena.giusto@aol.com (E.G.); arianna.rossi@istec.cnr.it (A.R.); giada.bassi@istec.cnr.it (G.B.); andrea.ruffini@istec.cnr.it (A.R.); monica.montesi@istec.cnr.it (M.M.)
2 Regional Centre of Advanced Technologies and Materials, Czech Advanced Technology and Research Institute, Palacký University Olomouc, 783 71 Olomouc, Czech Republic; ludmila.zarska@centrum.cz
3 Department of Chemistry, Maynooth University, Maynooth, Ireland; darren.beirne.2017@mumail.com
4 Department of Chemical, Biological, Pharmaceutical and Environmental Sciences, University of Studies of Messina, 98100 Messina (ME), Italy
5 Department of Neuroscience, Imaging and Clinical Sciences, University of Studies G. d'Annunzio Chieti-Pescara, 66100 Chieti (CH), Italy
6 Institute of Molecular and Translation Medicine, Faculty of Medicine and Dentistry, Palacký University in Olomouc, Hnevotinska 5, 779 00 Olomouc, Czech Republic
* Correspondence: diego.montagner@mu.ie (D.M.); vaclav.ranc@upol.cz (V.R.); silvia.panseri@istec.cnr.it (S.P.)
† These authors contributed equally to this work

AFM analysis.

The image and height profile of GO flakes in stock solution (Fig S1A and S1B, respectively) and GO flakes in supernatant (Fig S1C and S1D, respectively) were determined using AFM. The size of the flakes in the supernatant was significantly reduced compared to the stock solution.AFM was also used to analyze the number of GO flakes in 1 mL of supernatant. A 5 μL sample was spotted on a 0.5 cm radius mica substrate and after the sample dried, 10 random spots of 50x50 μm were scanned. The obtained images were firstly edited using Gwyddion software and then evaluated using ImageJ software. Based on the data obtained by analyzing all the obtained images, the number of GO flakes in 1 ml of supernatant was calculated and a size distribution graph was constructed (Fig S2). Data of 2142 GO flakes present in 10 scans were used for the evaluation. As mentioned 85% of the GO flakes were up to 130 nm in size, the remaining 15% of the flakes were in the size range of 131-1000 nm.

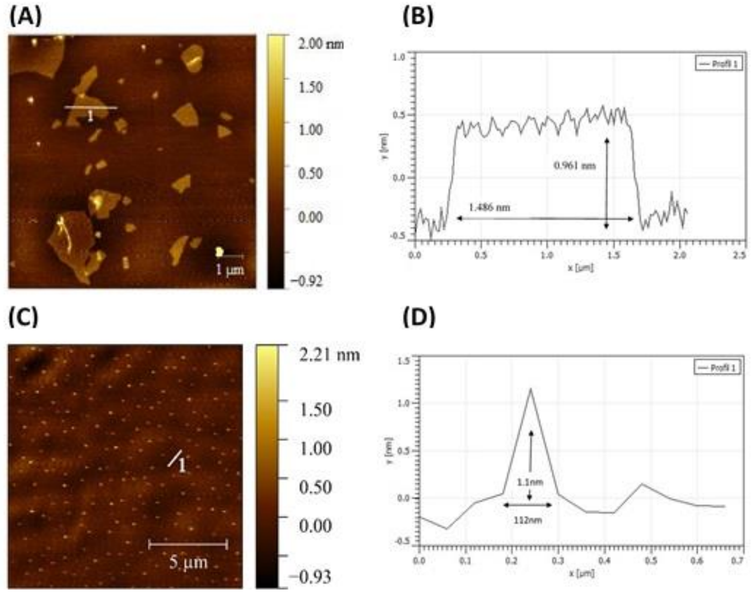

Figure S1. (**A**) The AFM image of GO flakes in stock solution and the height profile of the selected GO flake (**B**). The AFM image of GO flakes with reduced size (**C**), which was also demonstrated by the height profile of the selected GO flake (**D**).

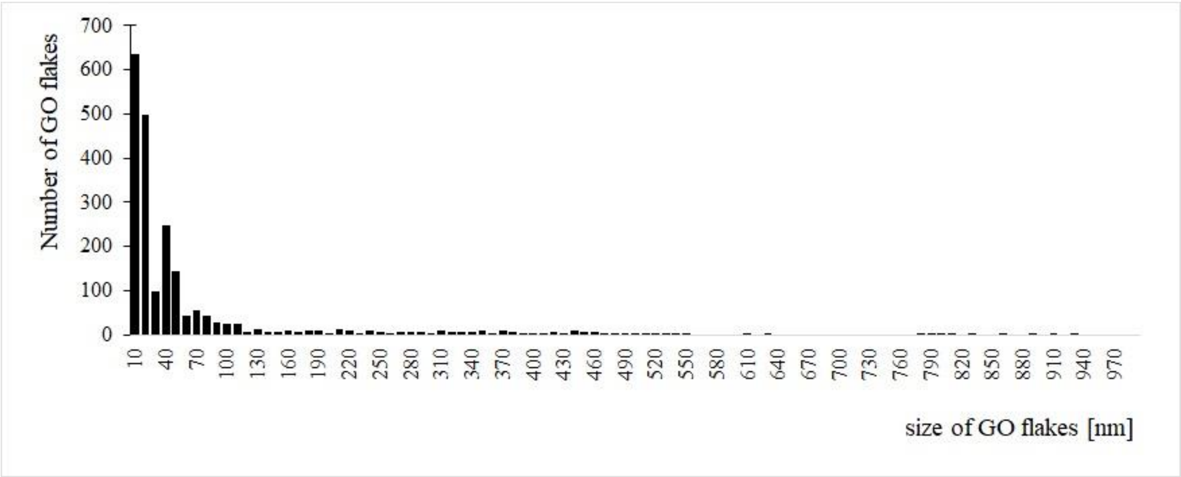

Figure S2. The size distribution of GO flakes in supernatant as observed from AFM images.

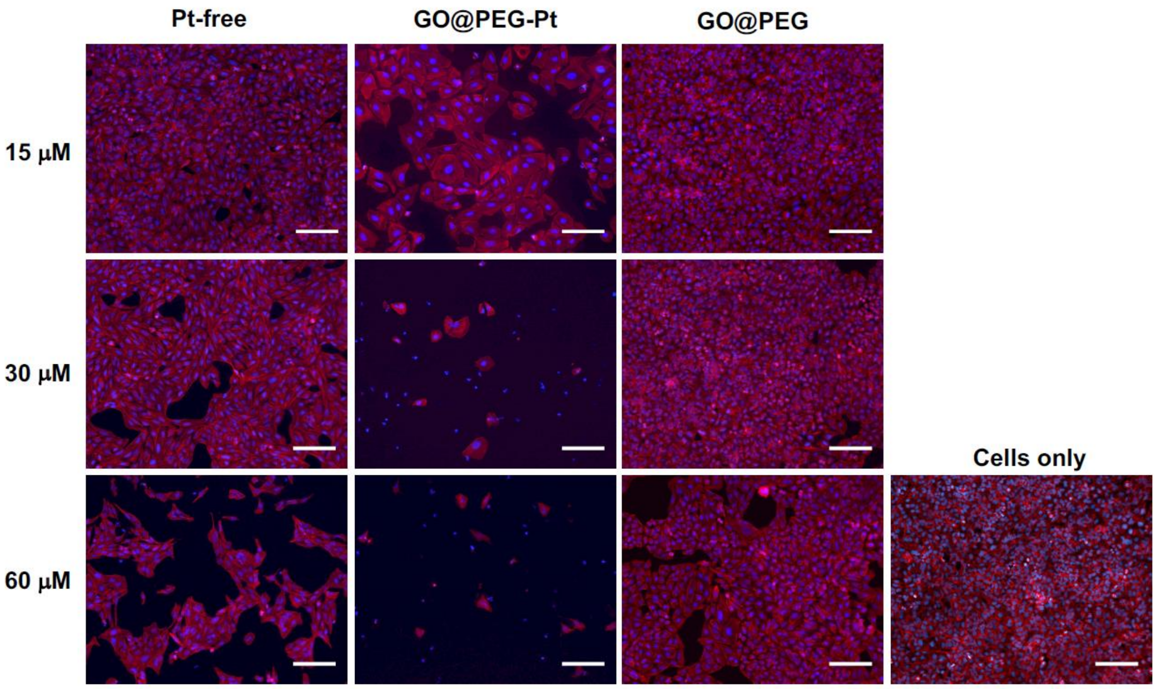

Figure S3. U2-OS cells' morphology evaluation at 72 hours. Phalloidin in red stains for actin filaments and DAPI in blue stains for cell nuclei. Scale bars: 100 μm.

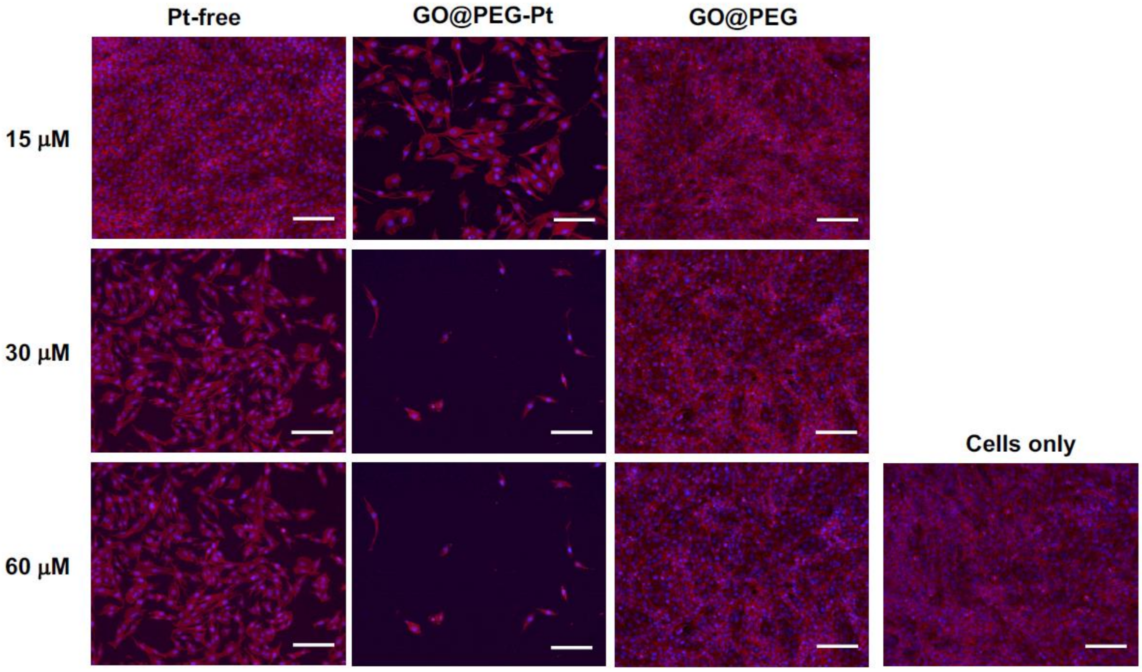

Figure S4. MG63 cells' morphology evaluation at 72 hours. Phalloidin in red stains for actin filaments and DAPI in blue stains for cell nuclei. Scale bars: 100 μm.

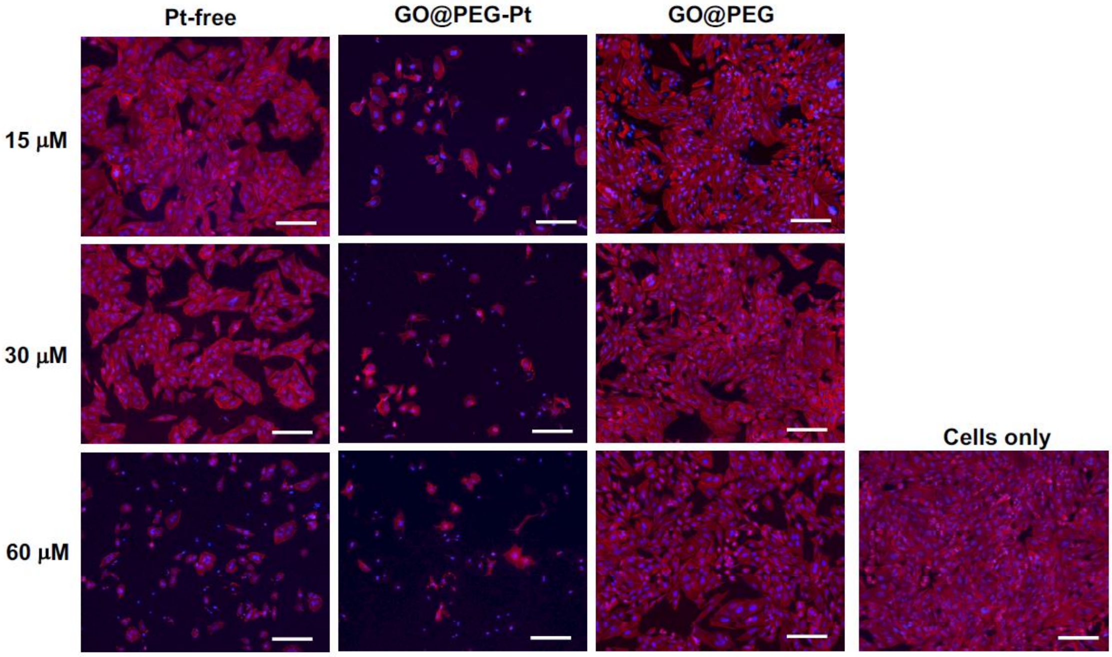

Figure S5. SAOS-2 cells' morphology evaluation at 72 hours. Phalloidin in red stains for actin filaments and DAPI in blue stains for cell nuclei. Scale bars: 100 μm.

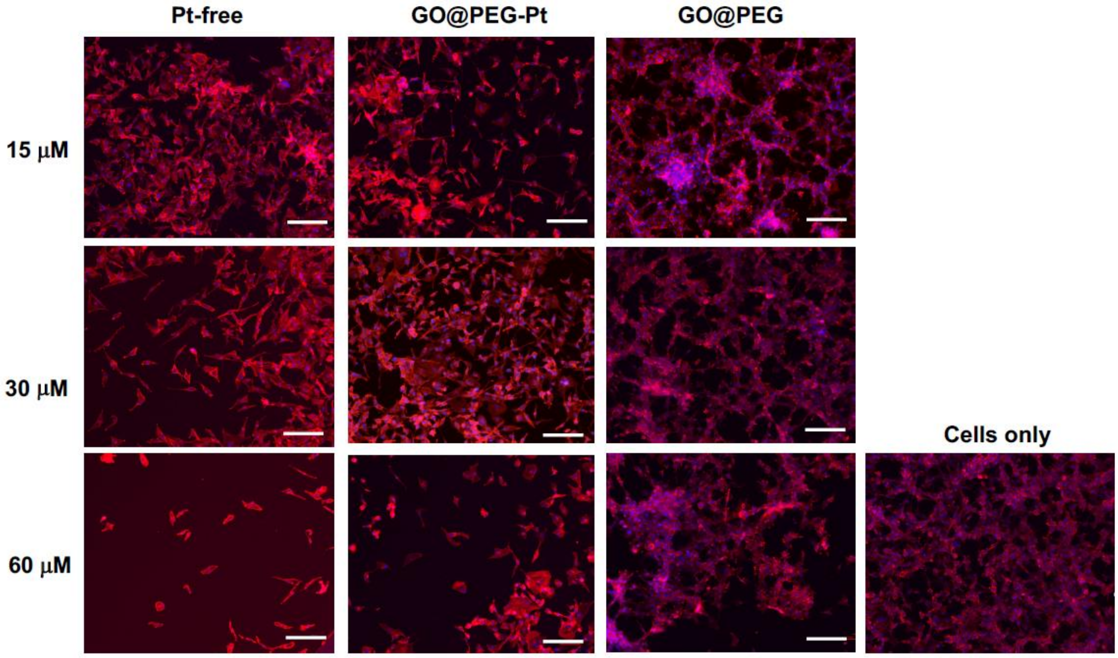

Figure S6. U87 cells' morphology evaluation at 72 hours. Phalloidin in red stains for actin filaments and DAPI in blue stains for cell nuclei. Scale bars: 100 μm.

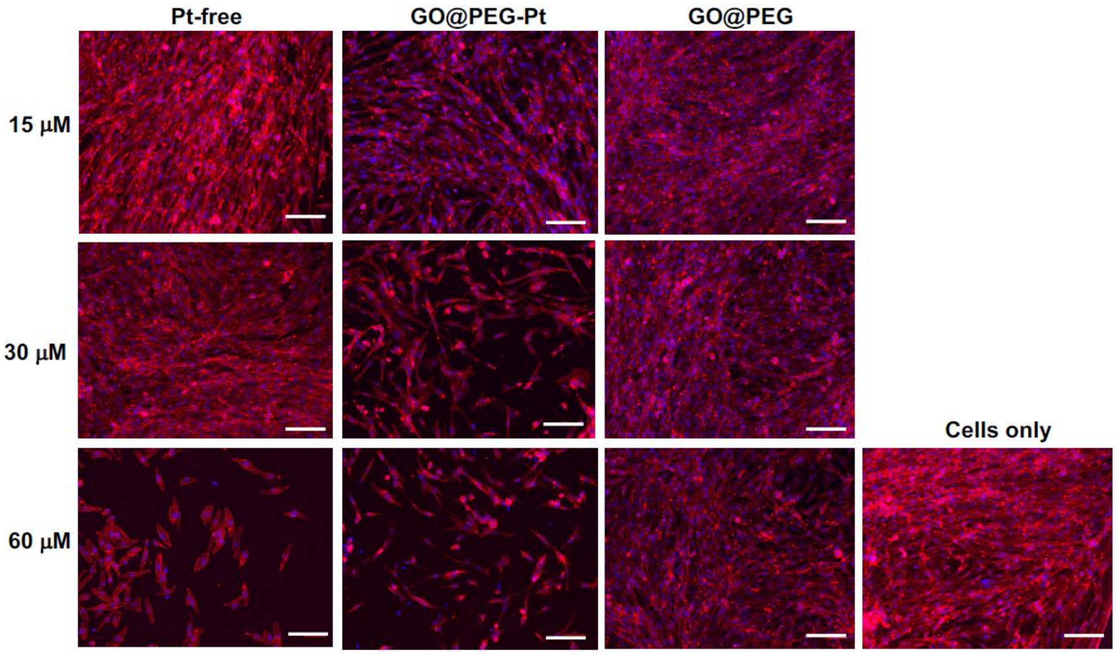

Figure S7. U118 cells' morphology evaluation at 72 hours. Phalloidin in red stains for actin filaments and DAPI in blue stains for cell nuclei. Scale bars: 100 μm.

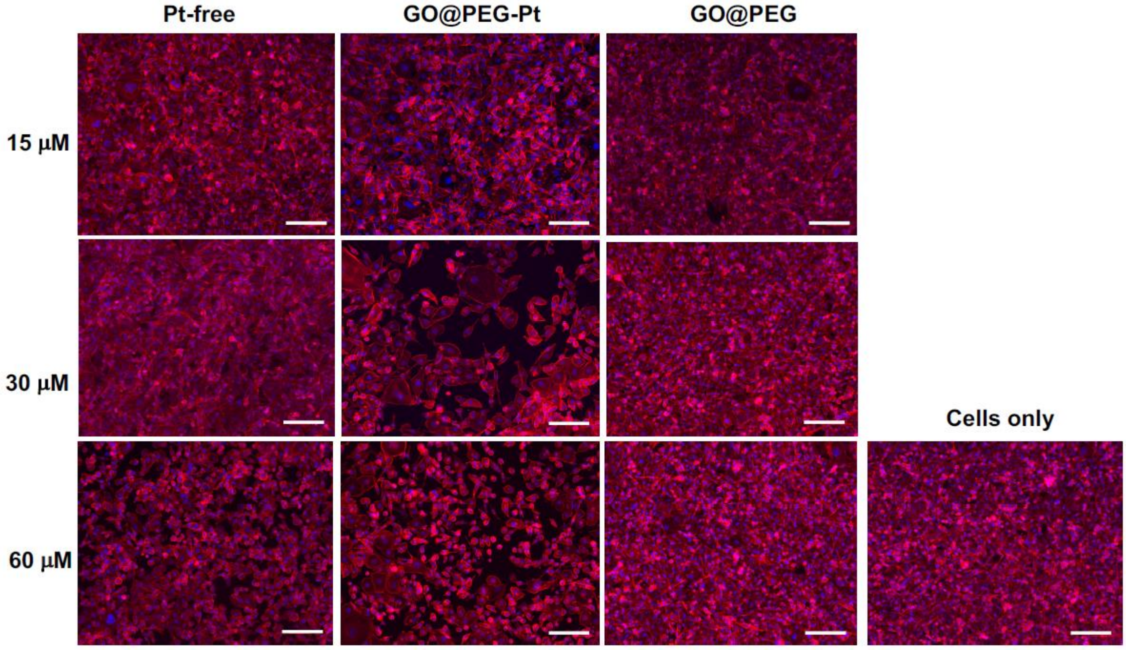

Figure S8. MDA-MB-231 cells' morphology evaluation at 72 hours. Phalloidin in red stains for actin filaments and DAPI in blue stains for cell nuclei. Scale bars: 100 μm.

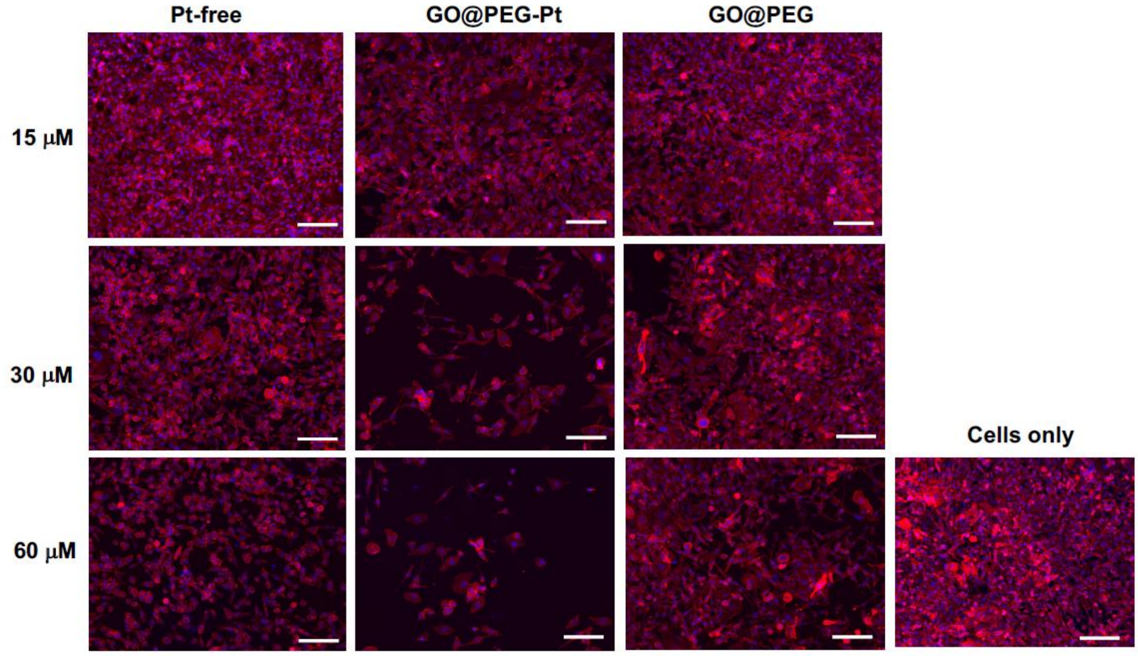

Figure S9. MDA-MB-468 cells' morphology evaluation at 72 hours. Phalloidin in red stains for actin filaments and DAPI in blue stains for cell nuclei. Scale bars: 100 μm.
